# Rapid Decrease in Fluoroquinolones Consumption following Implementation of a Simple Antimicrobial Stewardship Bundled Intervention in a University Hospital during the COVID-19 Pandemic

**DOI:** 10.3390/antibiotics12040694

**Published:** 2023-04-02

**Authors:** Raffaela Olivieri, Paola Vannini, Alice Corzani, Maria Teresa Bianco, Federico Franchi, Maria Grazia Cusi, Sabino Scolletta, Fabio Arena, Claudia Basagni, Roberto Gusinu, Mario Tumbarello

**Affiliations:** 1 Healthcare-Associated Infections Control Unit, Siena University Hospital, 53100 Siena, Italy; 2 Health Service Management Board, Siena University Hospital, 53100 Siena, Italy; 3 Pharmacy Unit, Siena University Hospital, 53100 Siena, Italy; 4 Anesthesia and Intensive Care Unit, Department of Cardiac, Thoracic, Vascular Sciences, Siena University Hospital, 53100 Siena, Italy; 5 Department of Medicine, Surgery and Neuroscience, University of Siena, 53100 Siena, Italy; 6 Microbiology and Virology Unit, Department of Innovation, Experimentation and Clinical Research, Siena University Hospital, 53100 Siena, Italy; 7 Department of Medical Biotechnologies, University of Siena, 53100 Siena, Italy; 8 Anesthesia and Intensive Care Unit, Department of Emergency-Urgency and Organ Transplantation, Siena University Hospital, 53100 Siena, Italy; 9 Department of Clinical and Experimental Medicine, University of Foggia, 71122 Foggia, Italy; 10 IRCCS Don Carlo Gnocchi Foundation, 50143 Florence, Italy; 11 Infectious and Tropical Diseases Unit, Department of Medical Sciences, Siena University Hospital, 53100 Siena, Italy

**Keywords:** antibiotic stewardship, daily defined dose, antimicrobial consumption, bundle, multidisciplinary team, data feedback, mandatory prescription motivation

## Abstract

Fluoroquinolones (FQs) represent an class of antibiotics of medical importance, but their use has been restricted due to their ecologic impact and associated side effects. The reduction of FQs use is an important goal of antimicrobial stewardship programs (ASP). This work describes an ASP focused on overall antibiotics and FQs consumption reduction. From January 2021, an ASP was implemented in a 700-bed teaching hospital. The ASP was based on: (i) antibiotics consumption monitoring system (DDD/100 bed days); (ii) mandatory antibiotic prescription-motivation (using a dedicated informatic format) with the goal of >75% of motivated prescriptions; and (iii) data feedback and training on FQs use indications. We evaluated the impact of the intervention on overall systemic antibiotics and FQs consumption according to the objectives posed by Italian PNCAR (National Action Plan on Antimicrobial Resistance). A decrease of 6.6% in antibiotic use was observed (2019 vs. 2021). Notably, the FQs consumption fell by 48.3% from 7.1 DDD/100 bd in 2019 to 3.7 DDD/100 bd in 2021 (*p* < 0.001). After six months of mandatory antibiotic prescription-indication, all units achieved the target set. The study suggests that a simple, bundled ASP intervention can be rapidly effective obtaining the objectives of PNCAR on the reduction of overall antibiotics and FQs consumption.

## 1. Introduction

Antimicrobial resistance, recently defined as a “silent pandemic”, remains one of the most urgent and serious global health challenges that can compromise the effectiveness of key medical interventions, such as cancer treatment and organ transplantation [1]. Antibiotics misuse is among the main drivers underlying the development of antimicrobial resistance, especially in healthcare setting. In fact, approximately the 75% of infections caused by antibiotic resistant bacteria in European countries in 2015 were hospital-acquired [2,3].

Antimicrobial stewardship programs (ASPs) play a critical role in improving the quality of antimicrobial use while optimizing patient outcome and minimizing the unintended consequences of antimicrobial misuse, including toxicity and resistance selection [4]. The term “antimicrobial stewardship” (AMS) has been defined by the ESCMID Study Group for AMS as “a coherent set of actions which promote using antimicrobials in ways that ensure sustainable access to effective therapy for all who need them” and the IDSA/SHEA recommendations for implementing an ASPs defined the best approaches for antibiotic stewardship programs to influence the optimal use of antibiotics [5,6].

Italy is among the European countries with high dissemination and consequent public health burdens of antibiotic-resistant bacteria [7]. The report from the ECDC visit to Italy on January 2017 confirmed that the antimicrobial resistance situation in Italian hospitals poses a major public health threat to the country, making it one of the Member States with the highest levels of resistance in Europe [8]. With the Agreement of November 2017, between the national government and the local regions, Italy adopted its first National Action Plan on Antimicrobial Resistance (PNCAR) for 2017–2020, extended to 2021 due to the COVID-19 pandemic [9]. The PNCAR represents a roadmap for implementing the Italian strategy against antimicrobial resistance.

In order to face the increasing resistance and spread of antibiotic-resistant microorganisms, the PNCAR provides specific objectives and the clearly stated roles of institutions for national coordination, both in the human medicine and the veterinary fields.

The Italian strategies in the hospital setting involve two objectives: (i) the reduction of systemic antibiotics consumption by more than 5% and (ii) the reduction of fluoroquinolones (FQs) consumption by more than 10%, in the 2016–2021 period.

Quinolones and FQs are synthetic antimicrobials that are highly effective in treating a wide range of infections and are among the most commonly prescribed antibiotics worldwide [10,11]. Although these drugs represent a class of antibiotics of considerable medical importance, their use as a first-line treatment has been restricted due to their high ecologic impact and the risk of clinically relevant side effects [12,13,14]. Nevertheless, these drugs remain among the most prescribed and used types of antibiotics in Italy. In 2019, quinolone antibacterial drugs were ranked as the third most frequently prescribed antibiotics in Italy, preceded only by penicillins and macrolides/lincosamides; however, as compared to 2018, a 3% reduction in cumulative antibiotic consumption was observed and the category that contributed most to this decline was that of FQs [15]. In fact, the EMA communication from 16 November 2018, which recommended restrictions in the use of FQs and quinolones, likely had a significant impact on the reduction of the consumption of FQs [16].

In this work, we described the implementation of a bundled antibiotic stewardship intervention with rapid effect on overall antibiotic use and, especially, FQs consumption. The intervention allowed us to achieve both objectives posed by the Italian PNCAR.

## 2. Methods

From 2019, at the Siena University Hospital, a large 700-bed tertiary-care teaching hospital, a multidisciplinary team, including medical doctors, specialists in infectious diseases, intensivists, clinical microbiologists, pharmacists, nurses, and statistics, was instituted. The team defines the policy for an appropriate use of antibiotics, meets several times a year, organizes an annual professional practices assessment, and suggests updates to local guidelines referring to hospital health management. The team coordinates an annual hospital point prevalence survey (PPS) of healthcare-associated infections and antimicrobial use according to the protocol proposed by ECDC (European Centre for Disease Prevention and Control) [10]. The team was named the AID Team (antimicrobial stewardship, infection prevention, and diagnostic stewardship) according to region legislation. The COVID-19 pandemic affected the activities of the AID Team in 2020, causing a delay in ASP implementation.

From January 2021, we implemented a bundled antibiotic stewardship program as a part of the AID Team activities, based on an (i) antibiotics consumption monitoring system; (ii) mandatory antibiotic prescription-indication; and (iii) feedback, audit, and educational interventions. The organizational structure of the hospital remained unchanged for the study period.

*Interventions*

### 2.1. Surveillance System of Antimicrobial Consumption

As a first step, an antibiotic consumption monitoring system was implemented with a retrospective data analysis on antimicrobial use for the years 2019 and 2020.

Antibiotics classification was carried out according to the Anatomical Therapeutic Chemical Classification code, ATC J01, and antibiotic usage data were obtained from the computerized database of the Pharmacy Unit after data analyst processing. The antimicrobial consumption was expressed as the defined daily dose (DDD), according to the World Health Organization’s definition as the assumed mean maintenance adult daily dose of an antimicrobial for 1 day of treatment [17].

We analysed systemic antibiotics consumption both cumulative and by antimicrobial class, expressed as DDD/bed days (DDD/100 bd). The number of bed days in 2019, 2020, and 2021 was 173.674, 145.823, and 161.505, respectively.

For consumption data visualization, we used a dedicated “heat map”, showing the magnitude of phenomena as colour intensities in two dimensions. The variation in colour by intensity gives obvious visual cues to the reader regarding how a phenomenon is clustered or varies over time, setting, and drugs.

The wards were grouped into five patient-care areas: medical, surgical, intensive care, COVID-19 (for 2020 and 2021 only), and maternity–paediatric areas. Stratified consumptions were calculated for each ward and referred “patient-care areas”.

The following indicators were selected according to PNCAR goals: total consumption of antibiotics (ATC code J01) and of FQs expressed in defined daily doses (DDD) per 100 bed days. Variations in consumptions were calculated using the formula [(2021 consumption/2019 consumption) − 1] × 100.

### 2.2. Mandatory Antibiotic Prescription-Indication Format in Electronic Medical Records

According to the hospital 2020 PPS data of healthcare-associated infections and antimicrobial use (data not shown), slightly more than half of antibiotic prescriptions were motivated in the medical records only.

Therefore, as a second step, in February 2021, the hospital management decided to set, as a 2021 outcomes-based incentive, at least 75% of the motivated antimicrobial prescriptions for certain critical wards. The targeted units were chosen among those with lower percentages of motivated prescriptions, as determined by 2020 PPS (Units A–G).

To facilitate the achievement of the objective, mandatory motivation registration for antimicrobial prescription in hospital electronic medical records by using a standardized prescription-format was introduced from June 2021. The format encourages physicians to select the motivation for the prescription from a predefined list whenever they prescribe an antimicrobial agent to be administered systemically (J01). The list of possible indications was taken from the Diagnosis website’s antimicrobial use list for empiric or targeted therapies of the European Protocol for Point prevalence survey. The list was amended to include medical and surgical prophylaxis [18].

### 2.3. Feedback, Audit, and Training

As a third step, during the first semester 2021, the health management conducted the systematic feedback on antimicrobial consumption to team AID and clinicians through audits, interviews, and trainings. The objective of these audits was to provide an update for healthcare workers on the advancements and preliminary results of the intervention.

During the audits, antibiotic consumptions were reviewed in order to compare antimicrobial use with other regional and national settings.

The interviews were conducted on the units with critical levels of FQs consumption (Units 1–3) and focused on indications for FQs use, the perception of the relationship between fluoroquinolone use and relevant side effects, and barriers to an intervention successfully restricting fluoroquinolone use. Interviews were conducted by the team AID manager, an infectious diseases specialist and a pharmacist, together with the hospital health director. The interviews were conducted via teleconferences or meetings.

We also provided educational session on antimicrobial stewardship within the institutional hospital annual training plan. In the first semester of 2021, a total of three editions focused on the rational use of antibiotics and aimed at medical doctor personnel were carried out.

#### Statistical Analysis

Simple linear regression analysis was used to assess the effect of the ASP on the trend of total antibiotics consumption and FQs consumption. Semi-annual trend changes in FQs consumption were tested using the semi-annual consumption data from 2019, 2020, and 2021 as time points.

A more detailed report with the analysis of consumption per month from 2019, 2020, and 2021 can be found in Figure S1a,b of the Supplementary Materials.

A *p* value of <0.05 was considered statistically significant. Statistical analyses were performed with the 9.3.1 version GraphPad Prism statistical package.

## 3. Results

### 3.1. Impact of Intervention on Antibiotic Use

Overall, antibiotic use decreased by 6.6% in the hospital, after ASP implementation, from 84.7 daily dose of antibiotics per 100 bed days (DDD/100 bd) in 2019 to 79.1 DDD/100 bd in 2021 (Table 1). Although not statistically significant in the linear regression analysis (*p* = 0.644), the reduction exceeded the target of a 5% decrease set by PNCAR. As shown in the heat map (Table 1), the most frequently prescribed antibiotics were the β-lactam antibacterials and penicillins (J01C).

**Figure 1:**
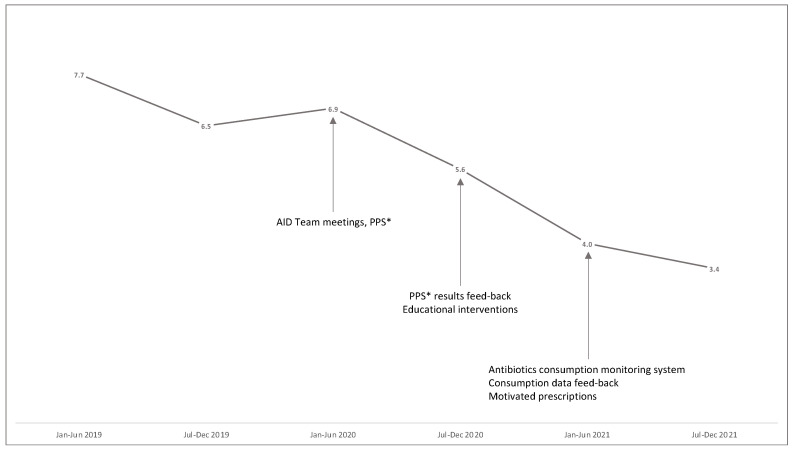
Evolution over time of FQs consumption (DDD/100 bd); * Point Prevalence Survey of healthcare-associated infections and antimicrobial use.

**Figure 2:**
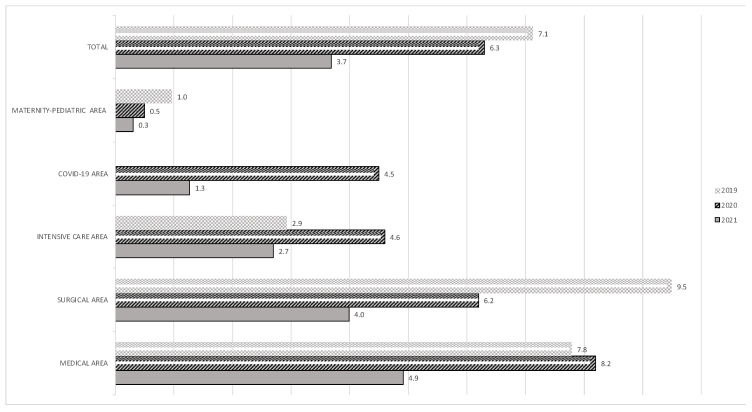
Fluoroquinolones consumption by patient-care area.

Analysing the data by antibiotic classes, we observed a reduction trend for 3rd and 4th generation cephalosporins (from 15.0 to 11.9 DDD/100 bd) and macrolides/lincosamides (from 7.2 to 4.4 DDD/100 bd). Notably, FQs consumption globally fell by 48.3% from 7.1 DDD/100 bd in 2019 to 3.7 DDD/100 bd in 2021 (*p* < 0.001) in the linear regression analysis. The target of a 10% reduction set by PNCAR was reached. The consumption of glycopeptides remained almost stable (from 6.4 DDD/100 bd to 7.5 DDD/100 bd). An increasing trend in the consumption of carbapenems was observed from 5.2 DDD/100 bd in 2019 to 7.1 DDD/100 bd in 2021 (37.8%). The antimicrobial use of the single antimicrobials is shown in Table 1. The trend in FQs consumption by semester is shown in Figure 1. Figure 2 shows fluoroquinolones consumption by patient-care area. The largest decrease in FQs consumption was observed in surgical, medical and maternity–paediatric patient-care areas from 2019 to 2021 (58.0%, 36.9%, and 68.4%, respectively).

We identified three wards with critical FQs consumption, two belonging to surgical areas (Units 1 and 3) and one to a medical area (Unit 2). The observed consumption ranged from 81.6 DDD/100 bd (Unit 3) to 32.0 DDD/100 bd (Unit 2) (Table 2).

After the intervention, the FQs consumption decreased in all three units, respectively, by 80.0%, (from 67.1 DDD/100 bd to 13.4 DDD/100 bd), 50.3% (from 32.0 DDD/100 bd to 15.9 DDD/100 bd), and 92.8% (from 81.6 DDD/100 bd to 5.9 DDD/100 bd), as shown in Table 2.

### 3.2. Impact of Intervention on Motivation of Antibiotic Prescriptions

After six months of mandatory antibiotic prescription-motivation system training, we performed the first evaluation (referring to the period June–December 2021).

The data analysis showed that the percentage of antibiotics prescriptions for which the motivation was documented in electronic medical records was over target (75% of the total antibiotics prescriptions) in all of seven targeted units (Table 3), with a global rate of motivated antibiotics prescriptions of 83.7%.

Analysing the data for selected indications by antibiotic class, it emerged that over the total amount of prescribed FQs (n. 234), 20.9% (49/234) was for surgical prophylaxis > 1 day (SP3), 14.5% (34/234) for symptomatic lower urinary tract infection (CYS), 13.7% (32/234) for pneumonia (PNEU) infections, and 9.8% (23/234) for gastrointestinal infections (GI) (Table 4).

## 4. Discussion

Continuous antibiotic consumption monitoring is the cornerstone of all AS strategies.

In our institution, in 2019 and 2020 before the intervention, the overall antimicrobial consumption was stable, with 84.7 and 84 daily doses of antibiotics per 100 bed days, respectively. In 2020, the most frequently prescribed antibiotics were the β-lactam antibacterials and penicillins (J01C), followed by the 3rd and 4rd generation cephalosporins, glycopeptides, and FQs, in line with the national data. In fact, in 2020, the penicillin combinations category (including beta-lactamase inhibitors) was the most prescribed in Italy (22.5 DDD/100 bd), accounting for a quarter of the total hospital consumption at the national level [15].

In 2020, the overall and FQs consumption in our hospital were already below the national data of 92.1 and 9.9 DDD/100 bd, respectively, but higher than those of the regional average for the same period (80.5 and 5.3 DDD/100 bd, respectively) [15,19]. In 2017, the World Health Organization (WHO) introduced the ‘Access, Watch, Reserve’ or AWaRe classification as a tool for improving the correct use of antibiotics; the group “Watch” includes antibiotic classes, such as FQs, that have higher resistance potential and includes antibiotics that are at a relatively high risk of the selection of bacterial resistance. The WHO recommends prioritizing this antimicrobial category as a key target of stewardship programs and monitoring [20].

Recently, quinolones have been subject to control by regulatory agencies for reasons of safety of use. The press release (12 April 2019) issued by the Italian Medicines Agency (AIFA) ended a lengthy process of reviewing safety data for quinolones and FQs at the EU level, announcing the withdrawal of some quinolones from the market and the implementation of use restrictions for all FQs. The procedure was initiated by the European Medicines Agency (EMA) in 2017, and based on the available evidence, the EMA concluded that the drugs under investigation were associated with serious, known, debilitating, long-term (months to years), and potentially irreversible side effects on one or more organs/systems [16,21].

In the EU/EEA hospital sector, there were statistically significant decreases in the mean 10-year trends for the consumption of quinolones (ATC group J01M) [22]. In Italy, FQs hospital consumption decreased significantly following the publication of the restrictive recommendations of the EMA and AIFA, dropping from 14.4 DDD/100 bd in 2018 to 9.9 DDD/100 bd in 2020 (−31.3%). Nevertheless, FQs remain the fourth most consumed class of antibiotics in 2020 with 9.9 DDD/100 bd, after Penicillin combinations (including beta-lactamase inhibitors) with 22.5 DDD/100 bd, cephalosporins with 17.5 DDD/100 bd, and macrolides with 16.2 DDD/100 bd, confirming the need for antimicrobial stewardship strategies targeting this class of drugs.

In fact, fluoroquinolone-resistance among clinically relevant pathogens continue rising. For example, the prevalence of FQs resistance in *Klebsiella pneumoniae* shows an increasing trend in Europe, rising from 25.3% in 2012 to 33.8% in 2020. In Italy, the situation is even more complicated, with an increase from 20% in 2009 to 52.4% in 2020. A similar trend was reported for *E. coli* [23].

For these reasons, we targeted our interventions to two indicators in order to achieve the objectives set by the PNCAR: (i) the reduction of the consumption of systemic antibiotics by more than 5% and (ii) the reduction of the FQs consumption by more than 10% in the 2016–2021 period.

In our institution, the decrease in FQ use was not balanced by an increased use of other antibiotic classes (with the exception of carbapenems), and during the study period, the overall antibiotic consumption in the whole hospital also decreased globally.

This project has allowed us to achieve the national objectives set by the PNCAR with a 48.3% reduction in the consumption of FQs (target 10%) and a 6.6% reduction in global consumption (target 5%).

During the interviews, it emerged that in some cases, professionals were not aware of the restrictions on FQs prescriptions. The core components of our ASP were the feedback of antimicrobial consumption data to providers and training on FQs-use indications as a persuasive approach, which made it possible to increase the level of awareness in prescribing physicians in conjunction with the mandatory documentation of the indication at the moment of antibiotic prescription. Through the documented indications system, we achieved a global rate of motivated antibiotic prescriptions of 83.7% compared to 51.1% in the 2020, as emerged from the Point Prevalence Survey of healthcare-associated infections and antimicrobial use.

A mandatory indication for antimicrobial agents is recommended by a number of organizations as a key element for implementing policies and interventions to improve antibiotic use [24]. This was shown to be associated with a reduction in antibiotics utilization and to help in the prescriptive appropriateness assessment [25,26,27].

Several studies have shown that the COVID-19 pandemic caused significant perturbations on antimicrobial consumption data, especially in the early periods of the pandemic [28]. For this reason, it is difficult to prove that the FQs consumption reduction was due only to the intervention itself rather than also due to the current pandemic’s influence (different case mixes, reduction in surgical interventions, etc.). The documented increase in the use of carbapenems should also be contextualized in the COVID-19 pandemic scenario. We will take these data into account when planning future interventions. 

Carbapenems and FQs are classified as reserve and watch groups, according to the World Health Organization (WHO) antimicrobial classification. The WHO target is that by 2023, at least 60% of all antimicrobial consumption should come from the access group (https://www.who.int/publications/i/item/2021-aware-classification, accessed on 25 January 2023) but the COVID-19 pandemic made it difficult to achieve this target [29].

The reduction in the FQs consumption achieved during the study period should be regarded as an important control measure for the consequent reduction of ecological pressure. FQs, in fact, have the potential to promote the development of antibiotic resistance and *Clostridioides difficile* infections [30,31]. Further analyses to evaluate the long-term impact of our study intervention would be of interest.

Our study has some limitations. First of all, the time of observation after the implementation of ASP may be too short to evaluate the overall impact of interventions. Another possible limitation is that, in the statistical analysis, we used linear regression analysis, which is widely used for its simplicity of comprehension, although for estimating trends over the time, the weighted least squares regression and robust regression methods would be the best options. In addition, we have not yet evaluated the prescriptive appropriateness, which is also an important target for the reduction of antibiotic consumption. This would require post-discharge antibiotic prescription data, which we could retrieve from the datasets of mandatory antibiotic prescription-indication formats in electronic medical records.

This study’s findings suggest that, in a broader context, policy makers should clearly set objectives at national and regional levels defining the responsibilities of the different institutions and the priorities to be adopted. Furthermore, the periodical monitoring of the achievement of defined goals is a fundamental tool in the improvement process, and it is necessary to implement AS programmes in all healthcare settings.

Based on results of our study, while planning interventions aimed at reducing in-appropriate antibiotic consumption, the antimicrobial stewardship team should focus primarily on (i) the systematic quantification of antibiotics consumption, global, and stratified per ward on a semi-annual basis; (ii) providing audits and feedback to healthcare workers; and (iii) the implementation of a system for mandatory antibiotic prescription-indication in medical records. At the same time, the aim is to increase the knowledge and the level of awareness of the appropriate use of antibiotics in all healthcare settings and for all healthcare staff, adopting both persuasive and restrictive interventions [31].

In conclusion, we demonstrate that a short-term antimicrobial stewardship bundle aiming at reducing the overall antibiotic consumption, with its main target being FQs prescription, can be effective in only a few months. Several other works have focused on this topic, but they were all based on long-term interventions [32,33].

## Figures and Tables

**Table 1:** Heat Map—antibiotic consumption in defined daily doses (DDDs)/100 bed days at the Siena University Hospital.

Antibiotic Class	2019	2020	2021
Total consumption	**84.7**	**84.0**	**79.1**
β-lactams and Penicillins	28.2	26.7	27.8
3rd and 4th generation cephalosporins	15.0	14.0	11.9
Glycopeptides	6.4	7.7	7.5
**Fluoroquinolones**	**7.1**	**6.3**	**3.7**
Macrolide and lincosamide	7.2	6.2	4.4
Carbapenems	5.2	6.0	7.1
1st and 2nd generation cephalosporins	3.5	3.8	3.3
Sulfonamides	3.2	3.2	2.8
Metronidazole	2.4	2.1	1.7
Aminoglycosides	1.7	2.0	2.6
Oxazolidinones	1.2	1.9	1.8
Phosphomycin	1.2	1.3	1.4
Lipopeptides	0.0	1.0	1.2
Tetracyclines	0.6	0.7	0.4
Ceftazidime-avibactam	0.0	0.6	0.7
Tygecicline	0.3	0.2	0.2
Colistin	0.1	0.1	0.3
β-lactams, other	0.2	0.1	0.1
anti-MRSA cephalosporins	0.0	0.0	0.1
Meropenem-vaborbactam	0.0	0.0	0.1
Cefiderocol	0.0	0.0	0.1

**Table 2:** Fluoroquinolones consumption in defined daily doses (DDDs)/100 bed days at critical FQs consumption units.

	Unit 1	Unit 2	Unit 3
2019	67.1	32.0	81.6
2020	63.8	29.2	24.9
2021	13.4	15.9	5.9
△ 2021–2019 (%)	−80.0%	−50.3%	−92.8%

Unit 1: ophthalmology; Unit 2: stem cell transplant; Unit 3: gynaecology.

**Table 3:** Successful motivated antimicrobial prescription units—June–December 2021.

	Indications (n.)	Prescriptions (n.)	Motivated Prescriptions (%)
Unit A	554	697	79.5
Unit B	451	578	78.0
Unit C	332	367	90.5
Unit D	257	325	79.1
Unit E	213	248	85.9
Unit F	209	233	89.7
Unit G	45	56	80.4

Unit A: medicine 1; Unit B: medicine 2; Unit C: general surgery; Unit D: pneumology; Unit E: thoracic surgery; Unit F: orthopaedic; Unit G: transplant surgery.

**Table 4:** Heat map—motivations for fluoroquinolones prescription (n. of prescriptions)—June–December 2021.

Antibiotic	SP1	SP2	SP3	MP	ASB	CYS	PYE	GUM	BAC	CSEP	SIRS	BRON	ENT	EYE	GI	IA	PNEU	SST-O	SST-SSI
Ciprofloxacin	6	11	34	8	3	26	3	3	5		1	5	1	2	23	9	9	2	6
Levofloxacin	2	6	14	1		8			2	4		6		3		1	21	1	1
Moxifloxacin			1										1	3			2		
Total	8	17	49	9	3	34	3	3	7	4	1	11	2	8	23	10	32	3	7

MP: medical prophylaxis; SP1: surgical prophylaxis single dose; SP2: surgical prophylaxis one day; SP3: surgical prophylaxis >1 day; ASB: asymptomatic bacteriuria; CYS: symptomatic lower urinary tract infection (e.g., cystitis); PYE: symptomatic upper urinary tract infection (e.g., pyelonephritis); GUM: prostatitis, epididymo-orchitis, STD in men; BAC: laboratory-confirmed bacteraemia; CSEP: clinical sepsis (suspected bloodstream infection without lab confirmation/results are not available, no blood cultures collected or negative blood culture), excluding febrile neutropenia; SIRS: systemic inflammatory response with no clear anatomical site; BRON: acute bronchitis or exacerbations of chronic bronchitis; ENT: infections of ear, nose, throat, larynx, and mouth; EYE: endophthalmitis; GI: gastrointestinal infections (e.g., salmonellosis, antibiotic-associated diarrhoea); IA: intra-abdominal sepsis, including hepatobiliary; PNEU: pneumonia; SST-O: cellulitis, wound, deep soft tissue not involving bone, not related to surgery; SST-SSI: surgical site infection involving skin or soft tissue but not bone.

## Data Availability

Not applicable.

## Supplementary Materials

The following supporting information can be downloaded at: https://www.mdpi.com/article/10.3390/antibiotics12040694/s1, Figure S1a: Linear regression analysis of total antimicrobial consumption; Figure S1b: Linear regression analysis of FQs consumption.
