# Supplementary material for: Rapid Decrease in Fluoroquinolones Consumption following Implementation of a Simple Antimicrobial Stewardship Bundled Intervention in a University Hospital during the COVID-19 Pandemic
Source: Antibiotics (Basel). 2023 Apr 2;12(4):694. doi: 10.3390/antibiotics12040694 (PMC10135293; doi:10.3390/antibiotics12040694)
Supplement: Supplementary file 1 [file antibiotics-12-00694-s001.zip › antibiotics-2275330-supplementary.pdf]

Figure S1 a. Linear regression analysis of total antimicrobial consumption.

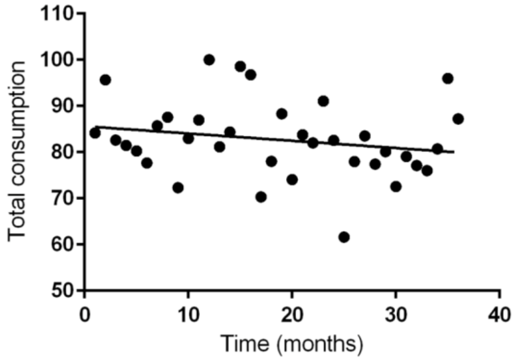

**Best-fit values**	
Slope	-0.1573 ± 0.1311
Y-intercept	85.63 ± 2.782
X-intercept	544.3
1/Slope	-6.356
**95% Confidence Intervals**	
Slope	-0.4240 to 0.1093
Y-intercept	79.98 to 91.29
X-intercept	213.4 to +infinity
**Goodness of Fit**	
R square	0.04063
Sy.x	8.172
**Is slope significantly non-zero?**	
F	1.440
DFn,DFd	1,34
P Value	0.2385
Deviation from horizontal?	Not Significant
**Data**	
Number of XY pairs	36
Equation	Y = -0.1573*X + 85.63

Figure S1 b. Linear regression analysis of FQs consumption.

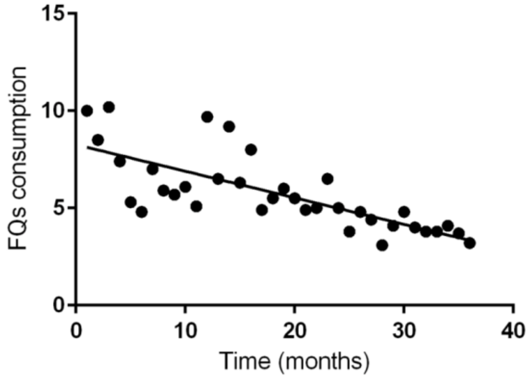

**Best-fit values**	
Slope	-0.1366 ± 0.02076
Y-intercept	8.266 ± 0.4404
X-intercept	60.51
1/Slope	-7.321
**95% Confidence Intervals**	
Slope	-0.1788 to -0.09439
Y-intercept	7.370 to 9.162
X-intercept	50.20 to 79.69
**Goodness of Fit**	
R square	0.5602
Sy.x	1.294
**Is slope significantly non-zero?**	
F	43.31
DFn,DFd	1,34
P Value	< 0.0001
Deviation from horizontal?	Significant
**Data**	
Number of XY pairs	36
Equation	Y = -0.1366*X + 8.266
